# Team Teaching Models in Primary Physical Education: Effects on Basic Motor Competencies and Self-Reported Physical Literacy

**DOI:** 10.3390/children12121595

**Published:** 2025-11-24

**Authors:** Gabriela Luptáková, Jaroslava Argajová, Tibor Balga, Dušana Augustovičová, Pavlína Sobotová, Gheorghe Balint, Branislav Antala

**Affiliations:** 1 Faculty of Physical Education and Sport, Comenius University in Bratislava, Nábr. arm. gen. L. Svobodu 9, 81469 Bratislava, Slovakia; jaroslava.argajova@uniba.sk (J.A.); tibor.balga@uniba.sk (T.B.); dusana.augustovicova@uniba.sk (D.A.); sobotova30@uniba.sk (P.S.); branislav.antala@uniba.sk (B.A.); 2 Faculty of Movement, Sports and Health Sciences, “Vasile Alecsandri” University of Bacău, Calea Marasesti, no. 157, 600115 Bacău, Romania; gbalint@ub.ro

**Keywords:** physical education, team teaching, motor competence, physical literacy, primary school

## Abstract

**Highlights:**

**What are the main findings?**

- No single configuration proved statistically superior across all groups; however, models including a PE specialist achieved the strongest within-group gains in both motor competence and self-perceived physical literacy.
- More frequent participation in organized out-of-school physical activity was significantly associated with higher basic motor competence.

**What are the implications of the main findings?**

- The Generalist Teacher + Coach model presents a paradox (improved motor skills but negatively impacted self-perceived physical literacy); therefore, PET-involved team teaching is recommended at the primary level for reliable gains in both objective motor skills and physical literacy self-perceptions.
- Objective motor competence and self-perceptions are positively correlated but capture distinct dimensions; effective physical literacy policy requires measuring both the objective skill and the affective domain.

**Abstract:**

**Background/Objectives:** To address the inconsistent provision of specialist physical education (PE) in primary schools, this study investigated the comparative efficacy of distinct team teaching configurations. The objective was to compare these instructional models’ impact on students’ basic motor competencies (MC) and self-reported physical literacy (PL). **Methods:** This cluster-randomized trial involved *N* = 266 students (grades 1–4) in Slovakia, assigned to one of five instructional models (PE teacher; GT+PET; PET+AT; GT+C; and GT+AT). The five-month intervention included two 45 min PE lessons weekly. Given the cluster design and non-normal data, the Wilcoxon singed-rank test and Kruskal–Wallis H-test were applied to assess the differences, and Cohen’s r was applied to determine effect size. **Results:** Comparative analysis showed no significant differences across teaching models for Self-Movement (*p* = 0.544), Object-Movement (*p* = 0.138), or PL (*p* = 0.219). Significant within-group MC gains were found in 4 teaching models, yet the practical effect size was generally weak to moderate (r ranging from 0.21 to 0.69). The strongest practical improvement was observed in the AT+PET Self-Movement group (r = 0.69). In contrast, the GT+PET configuration achieved no significant MC gain. For PL, only the AT+PET and GT+PET models showed significantly positive but moderate changes (r = 0.32 and 0.37). Conversely, the GT+C model resulted in a moderately significant decline in PL (Δ = −9.16, r = 0.43). A positive but practically weak correlation emerged between the MC subscales and PL (ρ ranging from 0.135 to 0.238, *p* < 0.05), with the highest limited association for Catching (ρ = 0.377, *p* < 0.01). The frequency of organized out-of-school physical activity was positively correlated with MC subscales (ρ = 0.195–0.282, *p* < 0.01) but not significantly correlated with PL. **Conclusions:** No single teaching model proved superior for improving overall motor competence or self-perceived physical literacy. While most effective configurations yielded moderate practical gains, the GT+C model presents a key paradox: while effective for objective skills, it proved detrimental to self-perceived physical literacy. These findings lead to explicit policy and implementation recommendations focused on strengthening collaborative instruction. Policymakers should consider strategies to support the integration of specialist PE teachers (PETs), such as establishing co-teaching as a recommended practice and allocating dedicated resources for funding and collaborative planning time to leverage the specialized knowledge they bring. Furthermore, schools are encouraged to focus on the effective implementation of PET-involved team teaching approaches (e.g., AT+PET and GT+PET). These models are not only effective but also support the co-professionalization of the generalist teacher, which is essential for ensuring that high-quality, evidence-based PE practices are consistently embedded.

## 1. Introduction

The concept of team teaching (also known as co-teaching or tandem teaching) is a collaborative instructional model in which two professionals instruct students in a single setting, based on mutual goals and shared responsibility [1,2,3]. Recognized as a component of educator professionalism, this partnership enhances instructional choices and promotes “activating learning” [4,5,6], offering richer learning experiences, increased curriculum access, and more personalized attention, fostering an inclusive culture [7,8,9,10,11,12]. The approach has gained significant traction in primary Physical Education (PE) internationally [13,14,15,16,17,18]. This shift is fueled by widespread concern that generalist teachers (GTs) are often inadequately equipped to deliver high-quality PE, citing insufficient knowledge of basic motor skills and inadequate training for progressive motor development [14,19,20,21,22,23,24]. This deficit is critical, as teacher competence is the strongest predictor of PE lesson quality and student outcomes [25,26]. Consequently, policymakers are increasingly turning to team teaching arrangements involving trained PE specialists (PETs) to ensure evidence-based pedagogical approaches [17,18,27,28,29], which in primary PE often takes the form of a “generalist plus one” [13,30].

Crucially, however, there is a methodological issue regarding the frequent conflation of “coach” and “specialist”, underscoring a vital distinction in expertise that affects instructional outcomes [31,32]. While perceptual evidence supports the value of specialist-involved team teaching, citing increased physical activity and student motivation [16,33,34], the overall effectiveness of various configurations remains inconclusive due to a persistent “empirical information deficit” [35,36]. Therefore, rigorous experimental designs are required to compare the impact of different expert-driven models on student development [37]. To fully address this gap, it is vital to establish the key outcome variables for student development. The first variable, basic motor competencies (MC), also known as Actual Motor Competence, refers to the ability to skillfully perform physical skills and movement patterns [38]. MC is critical, as low motor skill competence may make a child less inclined to participate in physical activities. However, MC alone is insufficient to describe lifelong engagement with physical activity, leading this study to also assess Physical Literacy (PL), which is a broader construct encompassing motivation, confidence, physical competence (MC), knowledge, and understanding to value and take responsibility for engagement in physical activities for life [38].

The self-reported PL measure is used to capture the subjective dimension of physical literacy, which includes Perceived Motor Competence alongside affective and cognitive domains. This relationship is complex and often weak, particularly at a young age, as the ability to accurately perceive motor competence increases with grade [38]. The low to moderate agreement between objective and perceived competence [39] highlights the necessity of measuring both domains. Specifically, we focused on the self-reported domains of PL because these dimensions—such as motivation and confidence—are hypothesized to be strongly and directly influenced by the instructional behavior and feedback provided within diverse team teaching configurations. It is acknowledged, however, that self-report measures are inherently limited by potential social desirability bias and subjective interpretation, necessitating their use as a complement to, rather than a replacement for, objective assessment.

**Figure 1:**
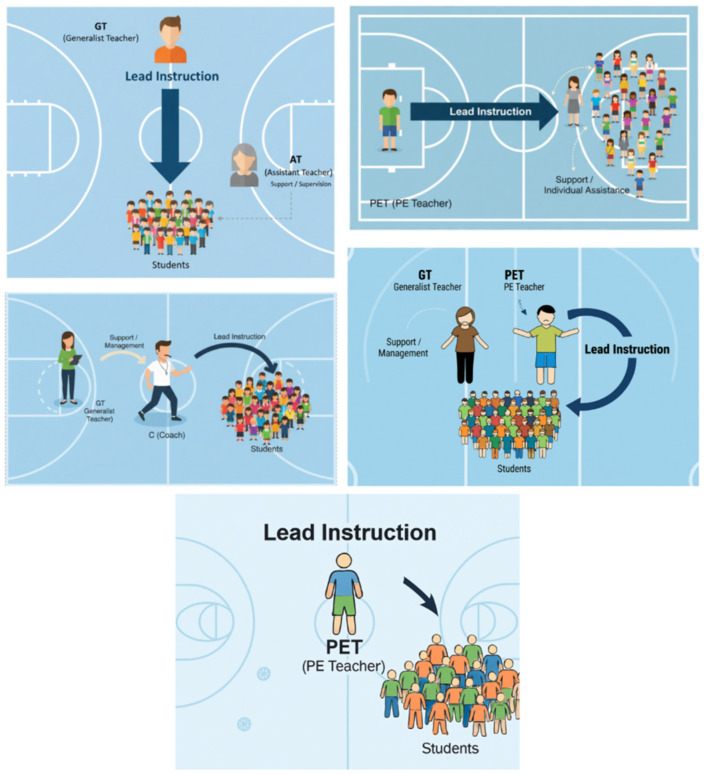
Schematic Representation of the Five Instructional Models Investigated in Primary Physical Education.

The current study addresses the critical gap by employing a comprehensive quasi-experimental design to compare a broader range of instructional models, including those with varying levels of specialization and co-teacher roles, against a single PE teacher control group. Teaching configurations are visualized in Figure 1.

The primary aim of this study was to investigate the comparative efficacy of distinct team teaching configurations and single PE teacher instruction on primary school students’ basic motor competencies (MC) and self-perceived physical literacy (PL). Furthermore, the study aimed to analyze the interrelationship between these two key outcome variables and investigate the associations between organized out-of-school physical activity and both MC and PL outcomes.

### Research Hypotheses

**H1** (Comparative Efficacy)**:** * Team teaching configurations involving a PE specialist will result in significantly greater gains in basic motor competencies and self-perceived physical literacy compared to the single PE teacher model and non-specialist models*.

**H2** (Variable Correlation)**:** * A significant positive correlation will exist between students’ objectively measured motor competencies and their self-perceived physical literacy following the intervention*.

**H3** (Covariate Association)**:** * Higher frequency and competitive involvement in out-of-school organized physical activity will be positively associated with higher scores in both motor competencies and self-perceived physical literacy.*

## 2. Materials and Methods

### 2.1. Study Design and Participants

This study utilized a quasi-experimental design where pre-existing classes were the unit of analysis, often referred to as a cluster-based approach. Pre-existing classes at three primary schools in the Bratislava region were selected based on availability and willingness to participate in the five-month PE intervention. Assignment to one of the five teaching configurations was performed at the class level to maintain the integrity of the instructional environment. The assignment of classes to one of the five configurations was conducted using simple random assignment. Class clusters were assigned without stratification. We confirm that no balancing (e.g., matching groups based on pre-test results, socio-economic status, or gender ratios) or explicit stratification was applied. This approach was selected to maintain the highest possible objectivity in the randomization process and avoid introducing researcher selection bias, which is a standard procedure for cluster-randomized trials where the intervention is delivered at the school level. Classes were pre-existing organizational units, and entire classes—not individual pupils—were allocated to instructional configurations to preserve ecological validity. This naturalistic cluster allocation avoided contamination between groups but inevitably introduced minor baseline heterogeneity.

Before commencement, written informed consent was obtained from the parents or legal guardians of all participating students (minors). The study was conducted in accordance with the Declaration of Helsinki and received ethical approval from the Ethics Committee of the Faculty of Physical Education and Sports, Comenius University in Bratislava, Slovakia (Approval no. 10/2024, dated 21 June 2024).

To investigate how instructional structure influences physical literacy and motor development, pre-existing classes were assigned to one of four distinct team teaching models or single PE teacher instruction. The five instructional configurations were structurally based on a Complementary Co-Teaching Model, where the expertise of each partner was intended to be leveraged to enhance instruction. The participant demographics for each configuration are detailed in Table 1, and the composition, qualifications, and intended roles are presented in Table 2.

The intervention utilized the standard state-mandated PE curriculum for the primary level in Slovakia. Crucially, the specific lesson content and pedagogical choices were not standardized, mandated, or monitored by the research team across the five instructional groups. The study aimed to investigate the efficacy of the different teacher configurations operating in their typical, naturalistic environment. This decision reflects a commitment to ecological validity, but it must be acknowledged that lesson content variability represents an uncontrolled confounding factor in the interpretation of the subsequent results.

Prior to the intervention, all participating teachers (PETs, GTs, Coaches, and ATs) were individually or collectively briefed by the research team regarding the study’s requirements. This communication focused on the research rationale, the importance of adhering to the intervention timeline, and general guidance on leveraging specialized expertise consistent with the intended roles in Table 2. This informal briefing served to orient teachers to the study’s parameters, but it did not constitute a formalized training session and did not include standardized role-playing or team-building exercises. Consequently, specific co-teaching roles were neither formally prescribed nor monitored, meaning that the instructional fidelity of the co-teaching process remains an unmeasured variable in this study.

### 2.2. Data Collection

Data collection occurred at baseline (T1) in October 2024, preceding the intervention, and post-intervention (T2) in April 2025, following the five-month experimental period.

Basic motor competencies were assessed at T1 and T2 using the Motor Competence Assessment (MOBAK) test instrument, which is designed to evaluate fundamental motor competencies and uses age-appropriate versions: MOBAK 1–2 (Grades 1–2) [40] and MOBAK 3–4 (Grades 3–4) [41]. The test measures two main categories: Self-movement (balancing, jumping, sidestepping/running, rolling) and Object movement (throwing, catching, dribbling, bouncing). All tests were administered by trained research assistants to ensure consistency. Scores for each competence area (Self-movement and Object movement) were initially recorded as raw sum scores (0–8 points) according to the MOBAK scoring protocol.

Self-reported physical literacy was assessed at T1 and T2 using the Physical Literacy Assessment for Youth-Self (PLAYself) questionnaire [42]. The PLAYself questionnaire was specifically chosen as a complementary measure to the objective MOBAK assessment. While MOBAK addresses the need for objective motor skill assessment (functionally equivalent to the physical competence dimension, such as PLAYfun), the PLAYself measure is essential for capturing the affective and psychological components of physical literacy (e.g., self-efficacy, motivation, and confidence). This dual-assessment strategy allows us to rigorously investigate the interplay between skill development and self-perception, which is one of the key aims of the study and central to determining the holistic efficacy of the different instructional models. The Physical Literacy Self-Description subscale, which measures a child’s self-efficacy related to physical activity participation, was used for analysis. The questionnaire was translated into Slovak using a rigorous forward–backward translation method to ensure conceptual equivalence. This process followed the established protocol by Chráska [43], involving (1) a forward translation into Slovak by two independent translators, (2) reconciliation into a unified version, (3) a blinded backward translation back into English, and (4) a final expert review to resolve any discrepancies. In line with standard psychometric principles for scale development, where α ≥ 0.70 is the accepted threshold for acceptable internal consistency in research settings, an internal consistency of α 0.70 was considered acceptable [43]. The internal consistency for the scale was α = 0.72. The questionnaire was teacher-administered for students in Grades 1 and 2, and self-administered for students in Grades 3 and 4.

To complement the results obtained in the school setting, Out-of-School Physical Activity (OOSPA) was assessed post-intervention (T2) using a context-specific questionnaire administered by teachers. This instrument was systematically derived from established regional and international physical activity recall protocols and tailored to the cultural and curricular context of Slovak primary education, thereby exhibiting strong ecological validity for this specific population. Questions focused on: (1) Frequency of Organized Physical Activity; (2) Competitive Involvement; and (3) Sport Type (individual or collective sports). These OOSPA data serve as a critical covariate in the statistical analysis, directly addressing our Hypothesis H3, and are essential for controlling the influence of external physical activity on both MC and PL outcomes to ensure the internal validity of our comparison between the teaching models.

### 2.3. Data Analysis

Data analysis was performed using IBM SPSS Statistics 21 for Windows. Given the fixed sample size (*N* = 266), post hoc sensitivity analysis using G*Power indicated 80% power (α = 0.05, two-tailed) to detect at least medium effects (r ≈ 0.30/η^2^ ≈ 0.06). Smaller effects may have remained undetected. All data distributions were tested for normality using the Shapiro–Wilk test. Since a lack of normality was observed in the change scores and the study used a quasi-experimental, cluster-randomized design, non-parametric tests and appropriate contextual reporting were employed. The statistical significance threshold was set at α = 0.05 (two-tailed). To control the family-wise error rate, *p*-values from pairwise post hoc tests were adjusted using the Holm–Bonferroni procedure. Missing data were <5% for all variables and handled by listwise deletion. Sensitivity checks with multiple imputation (m = 20) yielded comparable results.

The main objective of comparing the efficacy of the five instructional groups was addressed in two steps:

- Within-Group Change: To assess development within each configuration, the non-parametric Wilcoxon Signed-Rank Test was used to compare T1 and T2 scores for MOBAK Self-Movement, MOBAK Object-Movement, and Perceived Physical Literacy. The mean difference (Δ) and the 95% Confidence Interval (CI) for each difference were reported. Statistical significance for within-group change was determined if the 95% CI did not include zero and/or the Wilcoxon test yielded a *p* < 0.05.
- Between-Group Comparison: To statistically assess whether overall group differences in the T1 to T2 change scores were significant, a non-parametric Kruskal–Wallis H-test was conducted across the five instructional groups for each outcome measure. The Kruskal–Wallis test was chosen due to the lack of normality and the ordinal nature of the dependent variables.

To explore the relationship between the measured constructs and provide essential contextual data, Spearman’s rank-order correlations (ρ) were calculated:

- Correlations were calculated between children’s self-reported physical literacy and their objectively assessed motor competence (MOBAK subscale scores) at both initial (T1) and final (T2) testing to examine changes in self-awareness.
- Correlations were calculated between Out-of-School Physical Activity (OOSPA) variables (frequency, competitive involvement, and sport type) and the primary outcome measures (MOBAK subscales and self-reported physical literacy). This was carried out to quantify the potential influence of external activity on the study outcomes.

Effect sizes were reported for all inferential tests: for within-group changes, r = Z/√N; for Kruskal–Wallis tests, ε^2^ was computed. Effect sizes and Spearman correlation coefficients were interpreted using conventional cut-offs (0.10 = weak, 0.30 = moderate, 0.50 = strong), following Cohen’s guidelines [44].

## 3. Results

### 3.1. Changes in Basic Motor Competences by Instructional Group

Most importantly, the results indicated no statistically significant differences across teaching groups for Self-Movement (H(4) = 3.09, *p* = 0.544) or Object-Movement (H(4) = 6.96, *p* = 0.138). Although the Object-Movement test approached significance, these findings suggest that while within-group improvements occurred across several configurations, the comparative efficacy across the five groups was not statistically distinct.

**Figure 2:**
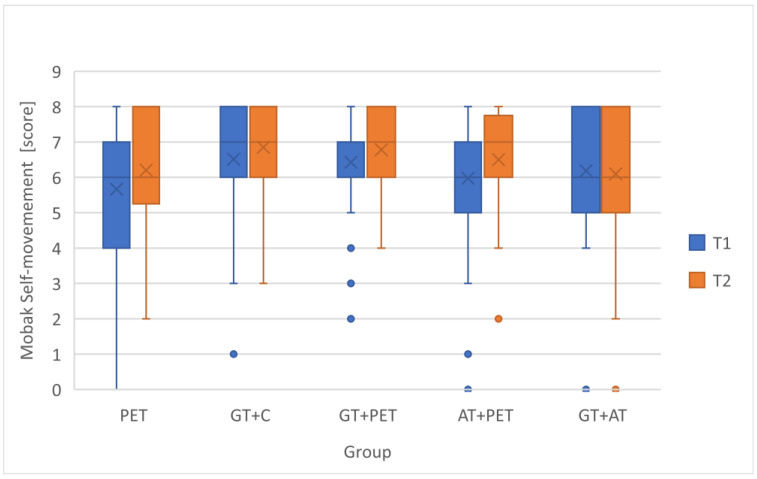
Within-group Changes in MOBAK Self-movement score between T1 and T2.

**Figure 3:**
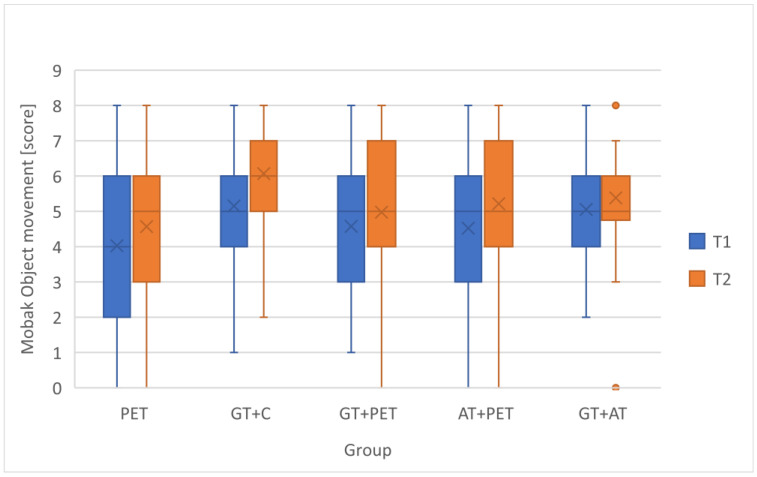
Within-group Changes in MOBAK Object-movement score between T1 and T2.

**Figure 4:**
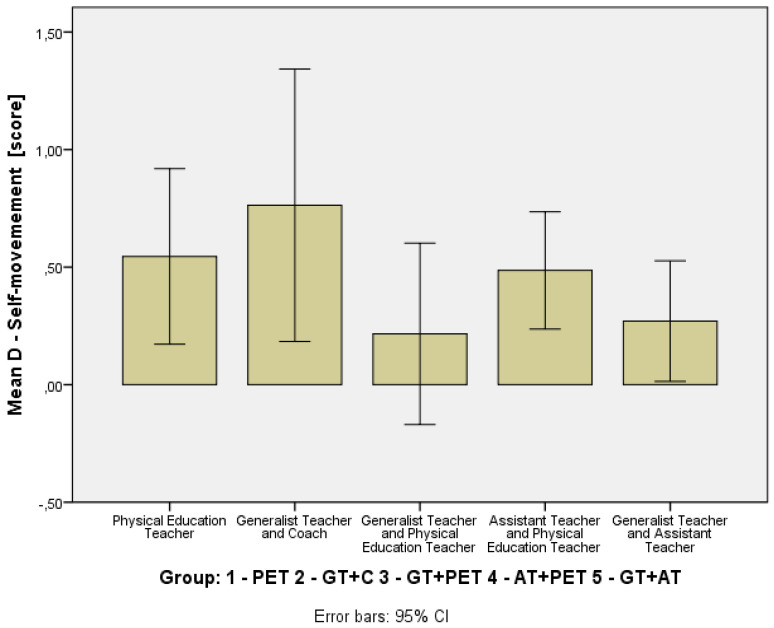
Mean Gain (T2–T1) in Basic Motor Competences: MOBAK Self-Movement Scores by Instructional Group.

**Figure 5:**
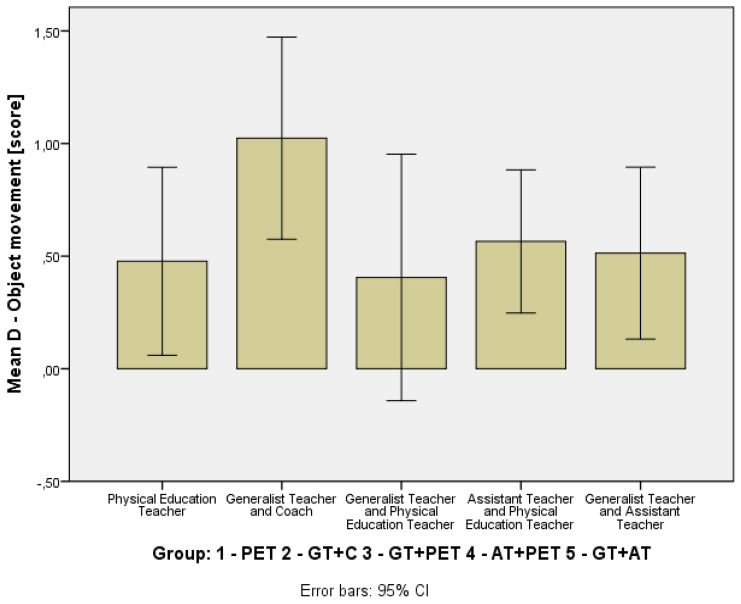
Mean Gain (T2–T1) in Basic Motor Competences: MOBAK Object-Movement Scores by Instructional Group.

To assess the students’ development in basic motor competencies, changes in the MOBAK Self-Movement and Object-Movement scores across different instructional groups were analyzed, as seen in Table 3. To visually depict distributional change, compact boxplots of pre- and post-scores for each instructional model were added (Figure 2 and Figure 3). In addition, Δ-change barplots illustrate the mean gain (T2-T1) for each outcome measure (MOBAK subscales) across the five instructional configurations (Figure 4 and Figure 5). Table 4 presents the *p*-values and effect sizes of within-group changes between T1 and T2.

In the Self-Movement subscale, the AT+PET group showed moderate improvement (Δ = +0.79, 95% CI = [0.45, 1.13]), which indicates a moderate effect (r = 0.32), closely followed by the GT+C group (Δ = +0.65, 95% CI = [0.18, 1.12]), also indicating a moderate effect (r = 0.30). Similarly, the PET-only (Δ = +0.40, *p* < 0.05, r = 0.38) and GT+AT (Δ = +0.30, *p* < 0.05, r = 0.43) groups showed statistically significant improvements with moderate effects, while the GT+PET group’s change (Δ = +0.19, CI crossing zero) was not statistically significant (r = 0.14), indicating a weak effect.

In the Object-Movement subscale, the PET-only group exhibited the largest increase (Δ = +1.07, CI = [0.19, 1.94]), a statistically significant improvement but with a weak effect (r = 0.28). The GT+C (Δ = +0.92, CI = [0.31, 1.54], r = 0.44), AT+PET (Δ = +0.53, CI = [0.04, 1.01], r = 0.30) and GT+AT (Δ = +0.43, *p* = 0.015, r = 0.40) groups showed statistically significant progress with moderate effects. In contrast, the GT+PET (Δ = +0.22, *p* = 0.224, r = 0.14), indicating a weak effect, had non-significant gains with confidence intervals that included zero.

**Figure 6:**
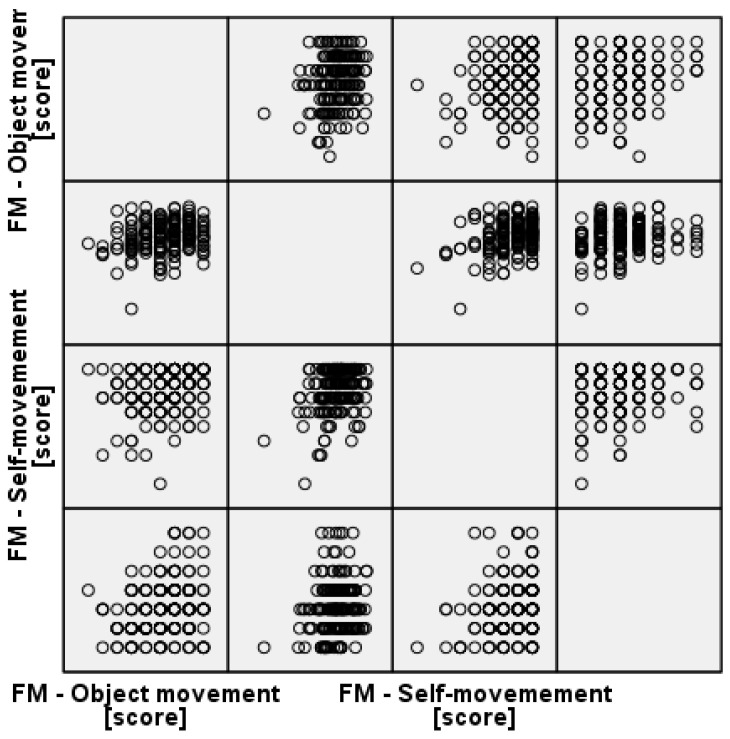
Correlation Matrix of Fundamental Movement (FM) Subscales: Object Movement vs. Self-Movement Scores.

Furthermore, Figure 6 suggests that the students’ ability to perform object-movement skills was only moderately or weakly correlated with their ability to perform self-movement skills within this specific sample, indicating that these subskills may be relatively independent or measured distinctly.

### 3.2. Changes in Self-Perceived Physical Literacy by Instructional Group

**Figure 7:**
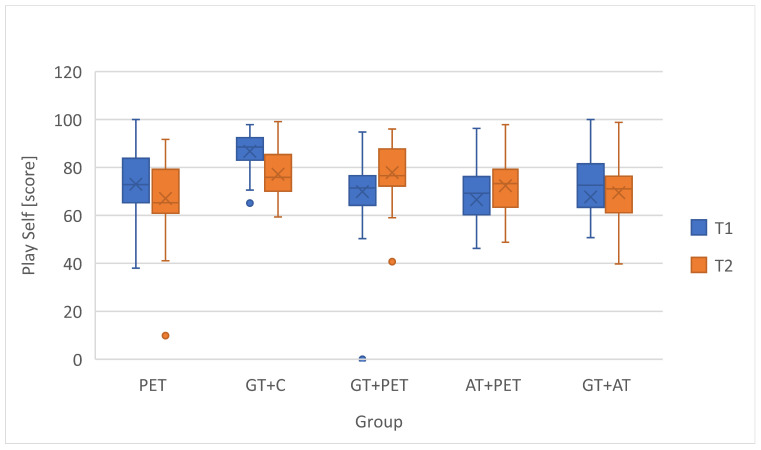
Within-group Changes in PLAYSelf score between T1 and T2.

**Figure 8:**
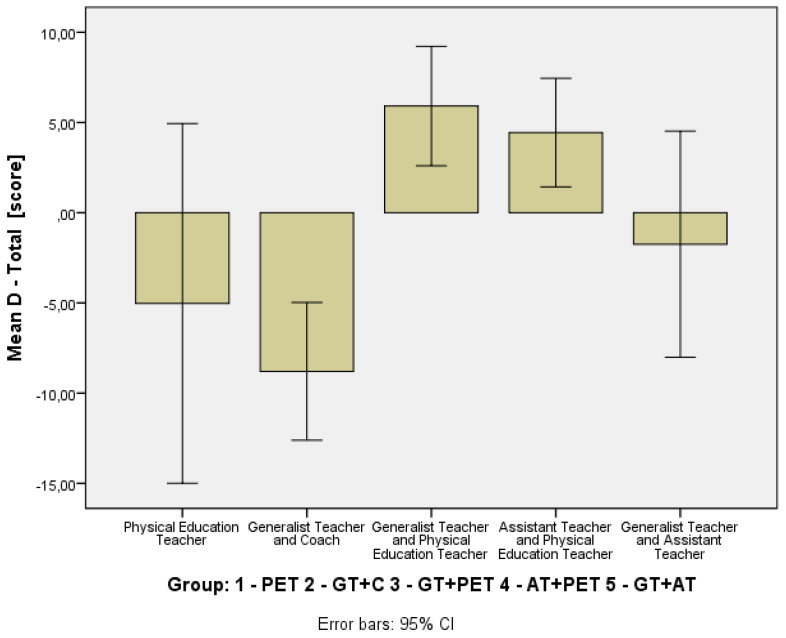
Mean Gain (T2–T1) in self-reported physical literacy: PLAYSelf Total Scores by Instructional Group.

To evaluate the effect of different instructional teams on the students’ self-perceived physical literacy, changes in PLAYself total scores were analyzed across five teaching configurations (Table 5). To visually depict distributional change, a compact boxplot of pre- and post-scores for each instructional model was added (Figure 7). The Δ-change barplot illustrates the mean gain (T2-T1) for PL across the five instructional configurations (Figure 8). Table 4 presents the *p*-values and effect sizes of within-group changes between T1 and T2 (see Section 3.1).

The GT+PET (Δ = +5.65, r = 0.37, *p* = 0.001) showed statistically significant gains with a moderate effect. The AT+PET (Δ = +5.82, r = 0.69, *p* = 0.002) groups showed positive gains in self-perceived physical literacy. This change was statistically significant and reflected a strong effect size, suggesting a strongly significant improvement in self-perceptions.

In contrast, the GT+C (Δ = −9.16, r = 0.43, *p* = 0.000) exhibited a statistically significant decline with a moderate negative effect size. The GT+AT group showed a non-significant increase of +2.72 (r = 0.05, *p* = 0.626), indicating a very weak effect, and PET-only non-significant decrease in PL (Δ = −3.42, r = 0.07, *p* = 0.563), also indicating a very weak effect.

Despite observable trends, a Kruskal–Wallis test comparing all five groups indicated that the differences were not statistically significant overall, H(4) = 5.75, *p* = 0.219. This suggests that while the use of specific instructional models (GT+PET and AT+PET) resulted in significant within-group gains in perceived physical literacy, there was no statistically significant difference in the magnitude of change when comparing the five models.

### 3.3. Associations Between Self-Perceived Physical Literacy and Motor Competence

**Figure 9:**
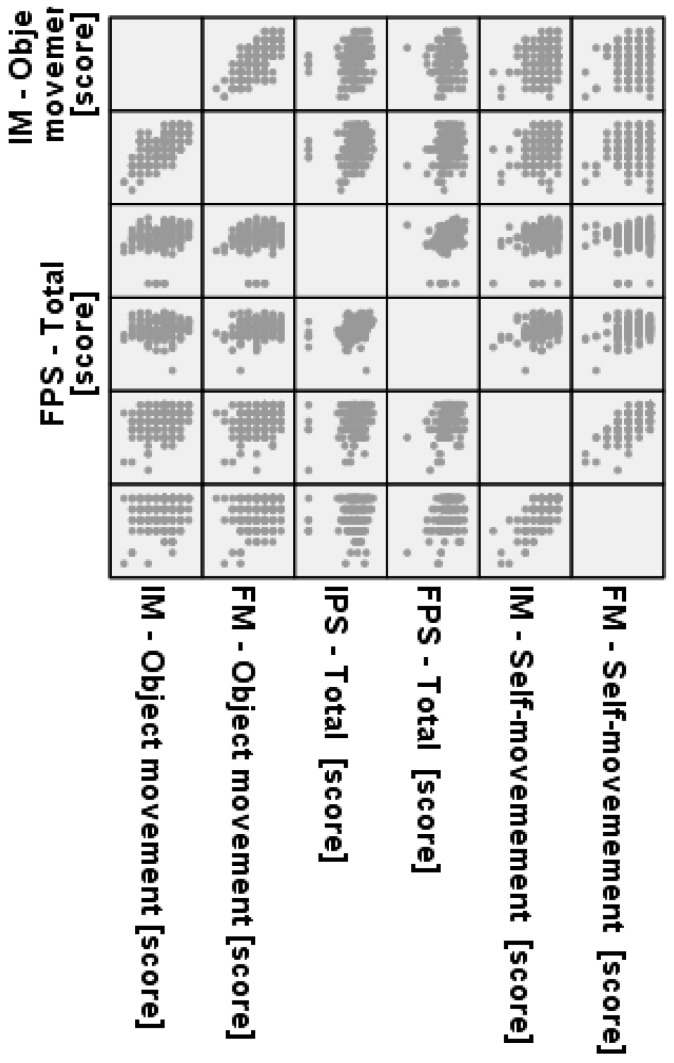
Correlation Between Objective Motor Competence (MOBAK) and Self-Reported Physical Literacy (PLAYSELF) at Pre- (T1) and Post-Intervention (T2).

To investigate the alignment between students’ subjective and objective competencies, Spearman’s rank-order correlations (ρ) were calculated between the PLAYself Total Score and the MOBAK subscales pre- and post-intervention (Table 6 and Table 7). A scatterplot illustrates the correlation between all relevant variables (MOBAK vs. PLAYSelf at T1 and T2) to visually support the findings of Hypothesis H2 (Figure 9).

At initial testing (T1), self-perceived physical literacy demonstrated weak but statistically significant positive correlations with objectively measured motor competence. As shown in Table 6, self-reported literacy was significantly associated with MOBAK Object-Movement (ρ = 0.183, *p* < 0.01) and MOBAK Self-Movement (ρ = 0.135, *p* < 0.05). At the individual skill level, the strongest, but still weak, association was observed between self-perception and catching (ρ = 0.223, *p* < 0.01), followed by running (ρ = 0.147, *p* < 0.05) (a weak correlation).

At final testing (T2), these relationships persisted, and in the case of self-movement, strengthened (see Table 7). The self-reported physical literacy maintained a weak, but significant positive correlation with MOBAK Object-Movement (ρ = 0.182, *p* < 0.05). Notably, the relationship with MOBAK Self-Movement became stronger and more significant (ρ = 0.238, *p* < 0.01) compared to the baseline, however, was still weak. Among individual skills, catching remained the strongest correlate (ρ = 0.377, *p* < 0.01) with a moderate level of correlation, suggesting that the children’s self-perception of literacy in this sample was most closely aligned with their proficiency in manipulative skills.

### 3.4. Associations Between Organized Out-of-School Physical Activity Participation and Basic Motor Competencies

Associations between organized out-of-school physical activity (OOSPA) and basic motor competencies are presented in Table 8. The analysis used bivariate correlations to provide contextual information regarding the students’ external physical activity levels. As part of the background assessment, children reported the type of organized sport they engaged in outside school. Overall, 43.0% participated in collective sports, 39.5% in individual sports, 1.2% in both, and 16.3% reported no participation. Frequency of participation in organized sport (OOSPA-Frequency) showed significant but weak positive correlations with both subscales of basic motor competence:

Object Movement (ρ = 0.282, *p* < 0.01), suggesting that students who engage more frequently in organized activity outside of school tend to display greater proficiency in skills like throwing, catching, dribbling, and bouncing.

Self-Movement (ρ = 0.195, *p* < 0.01), indicating a positive link between participation frequency and fundamental motor skills such as running, jumping, balancing, and rolling.

The variable Competitive Involvement was coded so that higher values (1 to 3) reflect less frequent participation in competitive settings (1 = Regularly; 3 = Never). Therefore, the negative correlation observed indicates that more frequent competitive involvement is positively associated with better outcomes. Specifically, higher competitive involvement (lower numerical code) was significantly, albeit weakly, linked to Object Movement (ρ = −0.145, *p* < 0.05). No significant association was found between competitive involvement and Self-Movement skills (ρ = −0.061).

The variable Sport Type (Collective, Individual, Both, or None) showed no statistically significant correlation with either the Self-Movement (ρ = 0.095) or Object Movement (ρ = 0.001) competencies.

### 3.5. Associations Between Organized Out-of-School Physical Activity Participation and Self-Reported Physical Literacy

Associations between the three dimensions of OOSPA and self-reported physical literacy are presented in Table 9. The results indicate that the relationship between organized sport participation and self-perception of physical literacy was weak, non-significant, and primarily driven by the frequency of participation.

Frequency of organized physical activity showed a weak positive association with self-perceived physical literacy (ρ = 0.133), which approached, but did not reach, statistical significance. This weak correlation suggests a marginal trend where students who participate more often in organized sports tend to report slightly higher self-efficacy related to physical activity.

There was no statistically significant association between competitive involvement and self-perceived physical literacy (ρ = −0.061), indicating a negligible association. Similarly, sport type (collective/individual) showed no statistically significant association with perceived physical literacy (ρ = 0.111), indicating a weak association.

In summary, none of the dimensions of OOSPA demonstrated a statistically significant correlation with self-reported physical literacy at the α < 0.05 level, suggesting that a child’s external involvement in organized sports was not strongly predictive of their self-reported physical literacy in this sample.

## 4. Discussion

### 4.1. Comparative Instructional Efficacy

The primary aim of this study was to assess the comparative efficacy of various team teaching configurations against single-teacher instruction on the development of basic motor competencies (MC) and self-reported physical literacy (PL). Contrary to our hypotheses that a specific co-teaching model would prove statistically superior, the main finding indicated no statistically significant difference in the magnitude of change among the five instructional configurations for either MC or PL. This null comparative finding suggests that over a standard five-month curriculum, the mere presence of an additional teacher or specialist, irrespective of their defined role, was not a sufficient condition to create a statistically distinct comparative advantage between the groups. This result aligns with the “empirical information deficit” highlighted in previous quantitative research [35,45], as a significant body of evaluative studies has similarly failed to establish a clear, statistically superior co-teaching model, particularly when assessing academic outcomes [37].

This outcome must be interpreted in light of the methodological challenges inherent to quasi-experimental designs in educational settings. As Veteska et al. [6] argue, the true impact of team teaching is determined primarily by how it is put into practice, rather than the theoretical configuration itself. Differences in teacher collaboration quality, fidelity of planning, and lesson-to-lesson instructional choices—all factors that differed across the five unique configurations—likely served as confounding variables. This underlying complexity made it difficult to detect a specific superior configuration, ultimately leading to the observed null comparative finding.

### 4.2. The Crucial Role of Specialist Expertise

While the analysis did not yield statistically significant differences between the five instructional configurations, the detailed descriptive data revealed a critical pattern: the largest and most consistent significant gains in basic motor competencies were confined to the configurations that included a certified PE teacher. Specifically, the PET-only group achieved the largest gains in Object-Movement, and the AT+PET configuration showed the largest team-based improvement in Self-Movement. The finding that PET involvement was the major driving factor behind students achieving statistically significant individual development directly supports the established literature that identifies perceived teacher competence as the strongest predictor of PE lesson quality [26]. The PET’s specialized training, which emphasizes correct performance and skill application [46], likely enabled them to provide the precise, evidence-based pedagogical approach necessary to move students beyond simple participation toward genuine motor skill mastery [28]. The ability of the single-teacher PET and the PET in the AT+PET model to generate superior within-group gains in MOBAK directly reinforces previous experimental findings where groups taught by specialized kinesiologists statistically outperformed control groups taught by generalist teachers [29].

In our study, the absence of statistically significant differences across groups for both subscales is a key finding. It suggests that the instructional structure (i.e., whether a Generalist Teacher, Assistant Teacher, or Coach was present) was secondary to the presence of quality pedagogical expertise. The certified PE teacher, regardless of their co-teaching partner, was consistently associated with the largest within-group improvements, reinforcing the notion that the quality and expertise of the instructor is the primary driver of motor competence development, rather than the mere organizational structure of the teaching configuration.

Therefore, despite the absence of a statistically superior model across all outcome measures, the data consistently indicate that specialist PE teacher expertise is the essential ingredient for generating statistically meaningful motor skill development. These results strongly endorse the current policy trends in various international contexts that mandate the inclusion of certified professionals in primary PE.

### 4.3. Differential Impact on Self-Perception

The results for self-perceived physical literacy (PL) demonstrated a complex differential impact. The configurations leveraging certified PE teachers—specifically AT+PET and GT+PET—both achieved significant positive gains in student self-efficacy. This success suggests that the blending of pedagogical and specialized PE expertise may effectively foster a competence-supportive environment, a key factor within Self-Determination Theory. Indeed, the adoption of motivational teaching behaviors is recognized as a critical determinant of students’ quality of experience in PE [47], and these PET-inclusive groups appeared successful in cultivating such a climate.

Conversely, the most complex finding was the GT+C paradox: this group achieved moderate objective gains in Object-Movement skills but experienced a significant decline in perceived physical literacy. This striking inverse relationship is likely attributed to the instructional style employed by the specialist coach, which typically prioritizes direct, technical correction and performance standards. Given the GT+C cohort’s young age (M = 6.58), this type of feedback likely accelerated the developmental shift toward a more realistic, performance-contingent assessment of their abilities, moving away from the inflated, non-contingent self-perceptions common in early primary grades [48]. While this process resulted in improved objective motor skills, the observed psychological deflation poses a risk, as high perceived motor competence is a potent, independent predictor of engagement in physical activity [39]. This underscores that simply increasing the expertise in the room is insufficient; the pedagogical approach must actively foster psychological needs through mastery-oriented feedback, reinforcing that instructional style, rather than just structure, has a crucial bearing on student motivation and self-perception [34,49].

### 4.4. Interpretation of the Association Between Self-Perceived Physical Literacy and Motor Competence

Another key finding was the weak correlation between students’ objectively measured motor competence (MOBAK) and their self-perceived physical literacy (PLAYself). Although the observed correlation was statistically significant at initial testing (T1), this weak alignment highlights the inherent challenges associated with measuring an abstract construct like physical literacy using self-report instruments in young populations.

The limited correspondence is typical in this age group and reflects both developmental and methodological limitations. Children in early primary grades often struggle to accurately interpret and report on abstract constructs like overall physical literacy, which is further complicated by the risk of social desirability bias where participants tend to over-report positive traits [48]. Furthermore, self-report measures like PLAYself, while crucial for capturing the affective dimension of physical literacy, inherently capture perceived competence rather than objectively demonstrated ability, leading to known issues with validity and reliability when compared to performance-based measures [38,39].

This weak initial alignment contrasts with studies suggesting a complete mismatch in young children [38,39]. Crucially, the relationship persisted, and in the case of Self-Movement, strengthened over the five-month intervention. This emergence of stronger alignment suggests that prolonged, structured physical education—regardless of the specific instructional model—began to facilitate a more accurate internalized self-assessment. Washburn and Kolen [38] support this interpretation, noting that children’s ability to accurately perceive their motor competence tends to improve with age and consistent exposure. The strengthening relationship reinforces that fostering accurate self-perception is a developmental process actively supported in educational settings [50].

The fact that the strongest moderate correlation at final testing was observed for catching suggests that Object-Movement skills lend themselves more readily to external, measurable feedback, helping students to gauge their performance relative to peers, a process vital for developing accurate self-perception [38]. Ultimately, our results contribute to the evidence base confirming that promoting both competence and accurate self-perception is essential for encouraging active lifestyles [39], while simultaneously underscoring the necessity for future research to address the methodological disconnect between objective performance and subjective self-report in young children.

### 4.5. Associations Between Organized Out-of-School Physical Activity and Motor Competence

The analysis of Organized Out-of-School Physical Activity (OOSPA) participation provided crucial contextual information regarding the external factors influencing motor development in this sample. This study highlights the fact that the higher the participation in organized sports outside of school, the better the overall basic motor competence. Furthermore, the outcomes indicate that more frequent competitive involvement is positively associated with better Object-Movement outcomes.

To synthesize the findings, a critical pattern emerged when comparing the effects of internal (school-based) versus external (OOSPA) factors. The primary finding—that there were no statistically significant differences in motor competence gains between the five instructional configurations—suggests a limit to the influence of the school-based instructional model alone. This is particularly relevant given that school physical education typically comprises only 2 × 45 min per week, a limited time window in which to drive profound, comparative changes in complex motor skills.

When this limited instructional time is juxtaposed with the finding that OOSPA frequency showed a significant positive correlation with both Object-Movement (ρ = 0.282, *p* < 0.01) and Self-Movement (ρ = 0.195, *p* < 0.01) skills, the data highlight the potentially greater role of external variables.

Therefore, the study implies that the sheer volume and consistency of practice derived from organized out-of-school physical activity might be a more potent, or at least an equally important, driver for developing basic motor competence than the specific school-based instructional configuration or type of teacher. While the quality of instruction (as seen in the PET-led descriptive gains) is vital, the limited time dedicated to PE in the curriculum means that the overall volume of practice outside of school may exert the dominant influence on skill acquisition for children in this age group.

### 4.6. Limitations and Future Directions

The interpretation of the current study’s findings, particularly the non-significant comparative effects, must be framed by several methodological limitations inherent to the quasi-experimental design and the ecological setting.

Firstly, the study employed a quasi-experimental, cluster-randomized design, meaning that students were not individually randomized but were nested within pre-existing classes, which served as the unit of intervention assignment. This non-independence of observations violates a key assumption of certain inferential statistics (such as the non-parametric Kruskal–Wallis test used for group comparisons) and may lead to an underestimation of standard errors and inflated Type I error rates. Consequently, our findings were interpreted conservatively. Future research should apply multi-level modeling to properly account for nested data (students within classes) and estimate random effects attributable to cluster variability.

Secondly, a critical limitation is the non-randomized nature of the cluster assignment, which resulted in groups that were not fully equivalent at the baseline, specifically concerning sample size and age. The instructional configurations varied substantially in group size, reducing statistical power for detecting effects in smaller cohorts. More importantly, the GT+C configuration was associated with students who were significantly younger (Mean Age: M = 6.58) compared to the remaining groups (M ≈ 7.7). This chronological age disparity introduced a developmental confound, particularly for the PL measure.

Thirdly, the lack of gender-specific analysis represents a limitation, as potential differential instructional impacts on boys’ versus girls’ outcomes may have been obscured by the pooled data. For instance, previous research suggests that gender may moderate the effect of instructional style on self-perception, with girls often being more sensitive to the type and quality of instructor feedback. Future studies should prioritize stratifying the analysis by gender to determine whether certain co-teaching configurations yield distinct outcomes for different sexes.

Fourthly, regarding assessment fidelity, all MOBAK assessments were conducted by a single, highly trained researcher to eliminate inter-rater variability. However, the lack of a formalized inter-rater reliability (IRR) check—such as parallel scoring by a second expert or video-based scoring—is a limitation. While this design choice ensured consistency (intra-rater reliability), it prevented external verification of the scoring fidelity and any potential drift in the researcher’s application of the rubric over the assessment period.

Fifthly, the assessment of self-perceived physical literacy relied on the self-report questionnaire. This tool is susceptible to social desirability bias and halo effects, where children may potentially over-report their confidence. This limitation is exacerbated by the age disparity in the GT+C group; their younger age corresponds to a developmental stage of potentially inflated self-assessments, which is a crucial explanatory factor for the inverse relationship observed in perceived physical literacy.

Sixthly, in the pursuit of ecological validity, the study deliberately opted not to standardize or monitor specific lesson content, pedagogical choices, or the fidelity of the co-teaching implementation. Consequently, the observed outcomes reflect the combined effect of the intended structural configuration and the uncontrolled contextual variability arising from the teachers’ planning quality, communication, and real-time execution of their defined roles. This lack of fidelity monitoring is a significant limitation and prevents a definitive isolation of the structural model’s direct impact. Compounding this issue, the voluntary nature of the teachers’ participation introduces a potential self-selection bias, where only highly motivated teachers—or those in schools already prioritizing PE—may have opted to participate. This restricts the generalizability of the findings to less motivated school environments. Future studies should incorporate direct behavioral observation to quantify the teaching style and quality of feedback provided within each configuration.

Additionally, the five-month (two 45 min lessons per week) intervention period may represent an insufficient time dose to elicit statistically significant comparative changes between the different instructional groups. This is especially relevant given the baseline variability and the complexity of developing both objective motor competence and PL simultaneously. A longer intervention duration or having more PE lessons per week is necessary to adequately assess the long-term, structural efficacy of these co-teaching models.

Finally, the assessment of Out-of-School Physical Activity (OOSPA) relied on a non-validated, context-specific questionnaire administered only at the post-intervention (T2) time point. While a validated tool would have been ideal, this specific questionnaire was retained in the main methodology because it captured ecologically relevant participation data essential to understanding the local contextual factors influencing skill development in this particular sample. However, the lack of prior psychometric validation (reliability and validity) is a clear limitation, and the OOSPA data should thus be interpreted conservatively. Furthermore, the single time-point assessment prevented its use as a true baseline covariate to statistically control for prior physical activity habits, which were shown to correlate significantly with motor competencies in the results.

## 5. Conclusions

This five-month intervention, comparing five distinct team teaching configurations, revealed no statistically significant differences in overall student development for either basic motor competence (MC) or self-perceived physical literacy (PL) across the instructional groups. This null comparative finding suggests that instructional structure alone may not yield a statistical advantage. However, analysis of within-group change scores indicated that configurations utilizing certified PE teacher expertise were the most successful in generating reliable gains in MC. The data further showed differential effects based on team composition, notably the significant decline in self-perceived PL observed solely within the Generalist Teacher + Coach configuration. Collectively, these findings suggest that the quality and expertise of the instructor remain the primary factor for meaningful motor skill development and positive self-perception. These results support the continued importance of involving certified specialist PE teachers in primary physical education instruction and underscore the value of ensuring that teacher training places sufficient focus on pedagogical approaches, such as competence-supportive strategies, to ensure that instruction promotes psychological well-being alongside technical skill acquisition.

## Figures and Tables

**Table 1:** Participant Demographics by Instructional Configuration.

Configuration Name	*n* (Students)	Mean Age (Years)	SD
Physical Education (PE) teacher	48	7.44	1.27
Generalist teacher + Coach	48	6.58	0.54
Generalist teacher + PE teacher	44	7.66	1.54
PE teacher + Assistant teacher	82	7.70	1.21
Generalist teacher + Assistant teacher	44	7.68	0.64

**Table 2:** Operational Definitions of Instructional Configurations.

Configuration Name	Teacher Composition	Key Teacher Qualifications	Intended Roles
PET	Single Certified PE Teacher	Master’s in PE; single-teacher control group.	PET (All instructional roles)
GT+C	Generalist Teacher (GT) + Sports Coach (C)	GT (Primary Ed Master’s); Coach (Sport Science/License; national program).	Lead: Coach (skill execution). Support: GT (classroom management/pedagogy).
GT+PET	Generalist Teacher (GT) + PE Teacher (PET)	GT (Primary Ed Master’s); PET (PE Master’s).	Lead: PET (motor skill development). Support: GT (pedagogy/class management).
AT+PET	Assistant Teacher (AT) + PE Teacher (PET)	AT (Pedagogical Sciences; after-school); PET (PE Master’s).	Lead: PET (motor skill development). Support: AT (management/individual assistance).
GT+AT	Generalist Teacher (GT) + Assistant Teacher (AT)	GT (Primary Ed Master’s); AT (Pedagogical Sciences; after-school).	Lead: GT (lesson delivery). Support: AT (supervision/behavior management).

**Table 3:** Changes in Basic Motor Competences by Instructional Group.

Teaching Model		Median	Mean	95% Confidence Interval for Mean		Std. Deviation	Min	Max	IQR
				Lower Bound	Upper Bound				
Self-movement [score]	Physical Education Teacher	0	0.4	−0.15	0.95	0.99	−1	2	1
	Generalist Teacher & Coach	1	0.65	0.18	1.12	1.16	−1	3	2
	Generalist Teacher & Physical Education Teacher	0	0.19	−0.26	0.63	1.23	−2	4	1.75
	Assistant Teacher & Physical Education Teacher	0.5	0.79	0.45	1.13	1.04	−1	3	2
	Generalist Teacher & Assistant Teacher	0	0.30	0.00	0.61	0.70	−1	1	1
Object movement [score]	Physical Education Teacher	1	1.07	0.19	1.94	1.58	−3	4	1
	Generalist Teacher & Coach	1	0.92	0.31	1.54	1.52	−2	4	2
	Generalist Teacher & Physical Education Teacher	0	0.22	−0.34	0.77	1.54	−2	5	2
	Assistant Teacher & Physical Education Teacher	0.5	0.53	0.04	1.01	1.47	−2	4	3
	Generalist Teacher & Assistant Teacher	1	0.43	−0.07	0.94	1.16	−2	3	1

Note. IQR = Interquartile Range.

**Table 4:** Within-group Statistical and Practical Significance in Motor Competence and Self-reported Physical Literacy.

	PET			GT+C			GT+PET			PET+AT			GT+AT		
	OM	SM	PS	OM	SM	PS	OM	SM	PS	OM	SM	PS	OM	SM	PS
Z	−2.293	−2.784	−0.578	−3.593	−2.241	−3.609	−1.217	−1.013	−3.252	−3.235	−3.508	−3.026	−2.441	−2.27	−0.487
*p*	0.022	0.005	0.563	0.000	0.025	0.000	0.224	0.311	0.001	0.001	0.000	0.002	0.015	0.023	0.626
r	0.28	0.38	0.07	0.44	0.30	0.43	0.14	0.14	0.37	0.30	0.69	0.32	0.40	0.43	0.05

Note. PET = Physical Education teacher, GT+C = Generalist teacher + Coach, GT+PET = Generalist teacher + Physical Education teacher, PET+AT = Physical Education teacher + Assistant teacher, GT+AT = Generalist teacher + Assistant teacher, OM = Object-movement, SM = Self-movement, PS = Self-reported physical literacy, Z = Wilcoxon Signed-Rank Test Z-score, *p* = statistical significance, r = Cohens r (effect size).

**Table 5:** Changes in Self-perceived Physical Literacy by Instructional Group.

Teaching Model		Mean	Median	95% Confidence Interval for Mean		Std. Deviation	Min	Max	IQR
				Lower Bound	Upper Bound				
PlaySelf [score]	Physical Education Teacher	−3.42	4.37	−18.14	11.29	26.58	−79.67	26.89	30.16
	Generalist Teacher & Coach	−9.16	−12.04	−13.50	−4.81	10.76	−28.41	17.26	12.77
	Generalist Teacher & Physical Education Teacher	5.65	3.83	1.88	9.43	10.47	−16.81	25.33	16.51
	Assistant Teacher & Physical Education Teacher	5.82	5.69	2.47	9.16	10.17	−15.11	36.70	13.68
	Generalist Teacher & Assistant Teacher	2.72	−1.56	−5.54	10.99	19.12	−22.26	68.93	21.48

Note. IQR = Interquartile Range.

**Table 6:** Associations Between Self-Perceived Physical Literacy and Motor Competence—Initial performance (T1).

Correlation Coefficient	Balancing [Score]	Rolling [Score]	Jumping [Score]	Running [Score]	Self-Movement [Score]	Throwing [Score]	Catching [Score]	Dribbling [Score]	Bouncing [Score]	Object Movement [Score]	PlaySelf—Total [Score]
Balancing [score]	1	0.099	0.159 *	0.171 **	0.450 **	0.105	0.165 *	0.11	−0.04	0.11	0.066
Rolling [score]	0.099	1	0.217 **	0.197 **	0.558 **	−0.001	0.108	0.111	0.083	0.099	−0.039
Jumping [score]	0.159 *	0.217 **	1	0.1	0.734 **	0.202 **	0.317 **	0.026	0.106	0.222 **	0.12
Running [score]	0.171 **	0.197 **	0.1	1	0.533 **	0.046	0.181 **	0.226 **	0.163 *	0.234 **	0.147 *
Self-movement [score]	0.450 **	0.558 **	0.734 **	0.533 **	1	0.172 **	0.307 **	0.166 *	0.11	0.265 **	0.135 *
Throwing [score]	0.105	−0.001	0.202 **	0.046	0.172 **	1	0.312 **	0.161 *	0.158 *	0.590 **	0.098
Catching [score]	0.165 *	0.108	0.317 **	0.181 **	0.307 **	0.312 **	1	0.169 **	0.251 **	0.630 **	0.223 **
Dribbling [score]	0.11	0.111	0.026	0.226 **	0.166 *	0.161 *	0.169 **	1	0.303 **	0.655 **	0.03
Bouncing [score]	−0.04	0.083	0.106	0.163 *	0.11	0.158 *	0.251 **	0.303 **	1	0.672 **	0.088
Object movement [score]	0.11	0.099	0.222 **	0.234 **	0.265 **	0.590 **	0.630 **	0.655 **	0.672 **	1	0.183 **
PlaySelf—Total [score]	0.066	−0.039	0.12	0.147 *	0.135 *	0.098	0.223 **	0.03	0.088	0.183 **	1

Note. * Correlation is significant at the 0.05 level (2-tailed). ** Correlation is significant at the 0.01 level (2-tailed).

**Table 7:** Associations Between Self-Perceived Physical Literacy and Motor Competence—Final performance (T2).

Correlation Coefficient	Balancing [Score]	Rolling [Score]	Jumping [Score]	Running [Score]	Self-Movement [Score]	Throwing [Score]	Catching [Score]	Dribbling [Score]	Bouncing [Score]	Object Movement [Score]	PlaySelf—Total [Score]
Balancing [score]	1	0.162 *	0.124	0.295 **	0.456 **	0.087	0.003	0.02	0.106	0.076	0.145 *
Rolling [score]	0.162 *	1	0.155 *	0.267 **	0.556 **	0.013	0.097	0.09	0.201 **	0.149 *	0.062
Jumping [score]	0.124	0.155 *	1	0.109	0.766 **	0.189 **	0.272 **	0.138 *	0.036	0.234 **	0.214 **
Running [score]	0.295 **	0.267 **	0.109	1	0.521 **	0.04	0.271 **	0.117	0.210 **	0.217 **	0.193 **
Self-movement [score]	0.456 **	0.556 **	0.766 **	0.521 **	1	0.146 *	0.261 **	0.157 *	0.134 *	0.256 **	0.238 **
Throwing [score]	0.087	0.013	0.189 **	0.04	0.146 *	1	0.262 **	0.155 *	0.202 **	0.644 **	0.038
Catching [score]	0.003	0.097	0.272 **	0.271 **	0.261 **	0.262 **	1	0.194 **	0.236 **	0.613 **	0.377 **
Dribbling [score]	0.02	0.09	0.138 *	0.117	0.157 *	0.155 *	0.194 **	1	0.263 **	0.596 **	0.018
Bouncing [score]	0.106	0.201 **	0.036	0.210 **	0.134 *	0.202 **	0.236 **	0.263 **	1	0.663 **	0.111
Object movement [score]	0.076	0.149 *	0.234 **	0.217 **	0.256 **	0.644 **	0.613 **	0.596 **	0.663 **	1	0.182 *
PlaySelf—Total [score]	0.145 *	0.062	0.214 **	0.193 **	0.238 **	0.038	0.377 **	0.018	0.111	0.182 *	1

Note. * Correlation is significant at the 0.05 level (2-tailed). ** Correlation is significant at the 0.01 level (2-tailed).

**Table 8:** Associations Between Organized Out-of-School Physical Activity Participation and Basic Motor Competencies.

Correlation Coefficient	Frequency of Organized Physical Activity [Scale 1–6]	Competitive Involvement: 1—Regularly, 2—Sometimes, 3—Never	Sport Type: 0—None, 1—Collective, 2—Individual, 3—Both	Self-Movement [Score]	Object Movement [Score]
Frequency of Organized Physical Activity [scale 1–6]	1	−0.539 **	0.394 **	0.195 **	0.282 **
Competitive Involvement: 1—Regularly, 2—Sometimes, 3—Never	−0.539 **	1	−0.277 **	−0.061	−0.145 *
Sport Type: 0—None, 1—Collective, 2—Individual, 3—Both	0.394 **	−0.277 **	1	0.095	0.001
Self-movement [score]	0.195 **	0.061	0.095	1	0.256 **
Object movement [score]	0.282 **	−0.145 *	0.001	0.256 **	1

Note. * Correlation is significant at the 0.05 level (2-tailed). ** Correlation is significant at the 0.01 level (2-tailed).

**Table 9:** Associations Between Organized Out-of-School Physical Activity Participation and Self-Perceived Physical Literacy.

Correlation Coefficient	Frequency of Organized Physical Activity [Scale 1–6]	Competitive Involvement: 1—Regularly, 2—Sometimes, 3—Never	Sport Type: 0—None, 1—Collective, 2—Individual, 3—Both	PlaySelf [Score]
Frequency of Organized Physical Activity [scale 1–6]	1	−0.539 **	0.394 **	0.133
Competitive Involvement: 1—Regularly, 2—Sometimes, 3—Never	−0.539 **	1	−0.277 **	−0.061
Sport Type: 0—None, 1—Collective, 2—Individual, 3—Both	0.394 **	−0.277 **	1	0.111
PlaySelf [score]	0.133	−0.061	0.111	1

** Correlation is significant at the 0.01 level (2-tailed).

## Data Availability

The original data presented in the study are openly available in Zenodo at DOI: 10.5281/zenodo.17474303.

## Abbreviations

The following abbreviations are used in this manuscript:

PE	Physical Education
PL	Self-reported Physical Literacy
MC	Basic Motor Competencies
PET	Physical Education teacher
AT	Assistant teacher
GT	Generalist teacher
C	Sports Coach
OOSPA	Organized out-of-school physical activity
MOBAK	Test battery of basic motor competencies
T1	Initial Testing (Time Point 1/Pre-intervention)
T2	Final Testing (Time Point 2/Post-intervention)

## References

[1] Cook L., Friend M. (1995). Co-teaching: Guidelines for creating effective practices. Focus Except. Child..

[2] Cook L., Friend M. (2004). Co-Teaching: Principles, Practices, and Pragmatics.

[3] Hargreaves A., O’Connor M.T. (2018). Solidarity with solidity: The case for collaborative professionalism. Phi Delta Kappan.

[4] Strogilos V., King-Sears M.E., Tragoulia E., Voulagka A., Stefanidis A. (2023). A meta-synthesis of co-teaching students with and without disabilities. Educ. Res. Rev..

[5] Abrami P.C., Bernard R.M., Borokhovski E., Waddington D.I., Wade C.A., Persson T. (2015). Strategies for teaching students to think critically: A meta-analysis. Rev. Educ. Res..

[6] Veteska J., Kursch M., Svobodova Z., Tureckiova M., Paulovcakova L., Ifenthaler D., Isaías P., Sampson D.G. (2022). Longitudinal Co-teaching Projects: Scoping Review. Orchestration of Learning Environments in the Digital World.

[7] Friend M., Cook L., Hurley-Chamberlain D., Shamberger C. (2010). Co-Teaching: An illustration of the complexity of collaboration in special education. J. Educ. Psychol. Consult..

[8] Baeten M., Simons M. (2014). Student teachers’ team teaching: Models, effects, and conditions for implementation. Teach. Teach. Educ..

[9] Cook S.C., McDuffie-Landrum K. (2019). Integrating effective practices into co-teaching: Increasing outcomes for students with disabilities. Interv. Sch. Clin..

[10] Gardiner W. (2010). Mentoring two student teachers: Mentors’ perceptions of peer placements. Teach. Teach. Educ..

[11] Simons M., Coetzee S., Baeten M., Vlerick P. (2020). Measuring learners’ perceptions of a team-taught learning environment: Development and validation of the Learners’ Team Teaching Perceptions Questionnaire (LTTPQ). Learn. Environ. Res..

[12] Strogilos V., King-Sears M.E. (2019). Co-teaching is extra help and fun: Perspectives on co-teaching from middle school students and co-teachers. J. Res. Spec. Educ. Needs.

[13] Jones L., Green K. (2015). Who teaches primary PE? Change and transformation through the eyes of subject leaders. Sport Educ. Soc..

[14] Dockerty F., Pritchard R. (2023). Reconsidering models-based practice in primary physical education. Education 3-13.

[15] Ní Chróinín D., O’Brien N. (2019). Primary school teachers’ experiences of external providers in Ireland: Learning lessons from physical education. Ir. Educ. Stud..

[16] Luptáková G., Balga T., Antala B., Cihová I., Argajová J., Dovičák M., Sližik M., Gurský T., Macháček J. (2025). Collaborative instruction with external providers in primary physical education: A three-year comparative analysis of generalist teacher perspective. Kinesiol. Slov..

[17] Luptáková G., Popeska B., Ristevska H., Balga T., Klincarov I., Antala B. (2025). Tandem teaching for quality physical education: Primary teachers’ preparedness and professional growth in Slovakia and North Macedonia. Educ. Sci..

[18] Klincarov I., Popeska B., Mitevski O., Nikovski G., Mitevska-Petrusheva K., Majeric M., Velickovska L.A. (2018). Tandem teaching in physical and health education classes from teacher’s perspective. Proceedings of the 3rd International Scientific Conference Research in Physical Education, Sport, and Health.

[19] Duncombe R., Cale L., Harris J. (2018). Strengthening ‘the foundations’ of the primary school curriculum. Educ. 3-13 Int. J. Prim. Elem. Early Years Educ..

[20] Spittle S., Spittle M., Encel K., Itoh S. (2022). Confidence and motivation to teach primary physical education: A survey of specialist primary physical education pre-service teachers in Australia. Front. Educ..

[21] D’Isanto T., D’Elia F. (2021). Body, movement, and outdoor education in pre-school during the COVID-19 pandemic: Perceptions of teachers. J. Phys. Educ. Sport.

[22] D’Isanto T., D’Elia F. (2021). Primary school physical education outdoors during the COVID-19 pandemic: The perceptions of teachers. J. Hum. Sport Exerc..

[23] Milić M., Radić Hozo E., Maulini C., De Giorgio A., Kuvačić G. (2022). What is the place of physical education among the teaching priorities of primary school teachers? An empirical study on importance, qualification and perceived teachers’ competence. Educ. Sci..

[24] Miholić S. (2017). Kinesiological Competences of Primary Education Teachers in the Republic of Croatia. Ph.D. Dissertation.

[25] Francesco C., Coco D., Frattini G., Vago P., Andrea C. (2019). Effective teaching competences in physical education. J. Phys. Educ. Sport.

[26] Petračić T. (2023). The Relationship Between Factors and the Quality of Physical Education Teaching in Primary School. Ph.D. Dissertation.

[27] Popeska B. (2022). Tandem Teaching in Physical Education: Current Experiences and Possibilities for Improvement.

[28] Aliberti S., Kaçurri A., Kasa A., Giardullo G. (2025). Stakeholders’ opinions on teaching movement education in primary schools: A cross-sectional study. J. Phys. Educ. Sport.

[29] Gerovac A., Emeljanovas A., Petrusic T., Novak D. (2025). Generalist vs physical education specialist elementary school teacher: Do teacher characteristics matter?. Kinesiol. Slov. Sci. J. Sport.

[30] Antala B. (2024). Didaktika Telesnej a Športovej Výchovy pre Základné a Stredné Školy: Vybrané Kapitoly (Didactics of Physical and Sports Education for Primary and Secondary Schools: Selected Chapters).

[31] Randall V. (2023). ‘We want to, but we can’t’: Pre-service teachers’ experiences of learning to teach primary physical education. Oxf. Rev. Educ..

[32] McEvilly N. (2022). What is PE and who should teach it? Undergraduate PE students’ views and experiences of the outsourcing of PE in the UK. Sport Educ. Soc..

[33] Balga T., Luptáková G., Cihová I., Dovičák M. (2024). Evaluating the impact of tandem teaching with coaches and generalist teachers on primary school students’ perceptions in physical education. Coll. Antropol..

[34] Domville M., Watson P., Richardson D., Graves L. (2019). Children’s perceptions of factors that influence PE enjoyment: A qualitative investigation. Phys. Educ. Sport Pedagog..

[35] Gökbulut Ö.D., Akçamete G., Güneyli A., Toper Ö. (2020). Impact of co-teaching approach in inclusive education settings on the development of reading skills. Int. J. Whole Sch..

[36] Qualls L.W., Arastoopour Irgens G., Hirsch S.E. (2025). Co-Teaching as a dynamic system to support students with disabilities: A case study. Educ. Sci..

[37] Luptáková G., Antala B., Seman F., Popluhárová M. (2025). Effects of teacher specialization on physical fitness and motor skills in tandem-taught physical education in elementary school. Zbornik Radova/Proceedings.

[38] Washburn R., Kolen A. (2018). Children’s self-perceived and actual motor competence in relation to their peers. Children.

[39] Chai H., Xue R., Yao L., Miao M., Han B. (2023). Configurations of actual and perceived motor competence among elementary school children in China: Differences in physical activity. Front. Public Health.

[40] Herrmann C., Seelig H. (2014). MOBAK–1: Motorische Basiskompetenzen in der 1. Klasse: Testmanual (MOBAK-1: Basic Motor Competencies in First Grade: Testmanual).

[41] Herrmann C., Seelig H. (2015). MOBAK–3: Basic Motor Competencies in Third Grade: Testmanual.

[42] Physical Literacy Canada (2023). PLAYself Workbook. https://physicalliteracy.ca/wp-content/uploads/2023/09/PLAYself_workbook_2023_EN_WEB.pdf.

[43] Chráska M. (2007). Metódy Pedagogického Výskumu—Základy Kvantitatívneho Výskumu (Research Methods in Pedagogy—Basics of Quantitative Research).

[44] Cohen J. (1988). Statistical Power Analysis for the Behavioral Sciences.

[45] King-Sears M.E., Jenkins M.C., Brawand A. (2020). Co-teaching perspectives from middle school algebra co-teachers and their students with and without disabilities. Int. J. Incl. Educ..

[46] Tsangaridou N., Charalambous C.Y., Kyriakides E. (2024). Preservice classroom teachers’ views and experiences of teaching physical education: Does taking a physical education specialization matter?. Eur. Phys. Educ. Rev..

[47] Franco E., Coterón J., Spray C. (2025). Antecedents of teachers’ motivational behaviours in physical education: A scoping review utilising achievement goal and self-determination theory perspectives. Int. Rev. Sport Exerc. Psychol..

[48] Harter S. (1999). The Construction of the Self: A Developmental Perspective.

[49] Monacis D., Annoscia S., Limone P., Colella D. (2023). Examining the effects of reproductive and productive teaching styles interventions on primary schoolchildren: What implications for physical education teachers?. Phys. Educ. Theory Methodol..

[50] Costa R.Z.F., Marques I., de Santo D.L., Medina-Papst J. (2019). Relationship between children’s competence self-perception, academic performance and motor performance. J. Phys. Educ..

